# The hypothalamic–pituitary–thyroid axis, depression and risk of suicide: commentary, Luo et al

**DOI:** 10.1192/bjo.2024.750

**Published:** 2024-12-04

**Authors:** Charles B. Nemeroff

**Affiliations:** Department of Psychiatry and Behavioral Sciences, The University of Texas at Austin Dell Medical School, Austin, Texas, USA

**Keywords:** Depression, thyroid, mood disorders

## Abstract

There is a considerable literature on the relationship of thyroid function with risk of depression and responsiveness to depression treatment. This literature is briefly reviewed here, followed by a focus on the incremental advance provided by the findings of Luo et al on autoimmune thyroiditis and suicide attempts.

Similar to the other neuroendocrine axes, the hypothalamic–pituitary–thyroid (HPT) axis is organised hierarchically; at its apex is a tripeptide thyrotropin-releasing hormone (TRH), which is released from hypothalamic neurons projecting to the primary plexus of the hypothalamic–hypophyseal portal system in the median eminence and transported vascularly to the anterior pituitary gland, where it causes the immediate release of thyroid-stimulating hormone (TSH), also known as thyrotropin, from thyrotroph cells. TSH, in turn, is released from the adenohypophysis into the general circulation and acts on the thyroid gland to release the two thyroid hormones, triiodothyronine (T_3_) and thyroxine (T_4_). The availability of circulating thyroid hormones is regulated by the existence of thyroid hormone-binding proteins. The thyroid hormones feed back to the anterior pituitary thyrotrophs and to TRH-containing neurons in the brain to regulate HPT axis activity. In addition, there are thyroid hormone receptors for T_3_ not only in these sites but in higher brain regions as well. Thyroid hormone availability in the central nervous system is also regulated by a thyroid hormone transporter protein, transthyretin.

This summary of thyroid axis physiology is relevant because virtually every component of the HPT axis has been scrutinised and found in most studies to be significantly altered in a sizable minority of patients with major depression. Before reviewing those findings and those of Luo et al,^1^ it is, however, important to point out the high rate of depression in patients with primary hypothyroidism, first reported more than two centuries ago.^2^

What are the reported alterations in the HPT axis in patients with major depression?^3^ In most studies, there were higher rates of hypothyroidism in depressed patients, including not only those with reduced circulating thyroid hormones and elevated TSH (primary or grade I hypothyroidism) but more subtle forms, including those with normal peripheral thyroid hormone levels and elevated TSH and those with normal levels of both TSH and thyroid hormones but with the presence of anti-thyroid antibodies, i.e. anti-thyroid peroxidase (TPOAb) and anti-thyroglobulin antibodies (TgAb), so-called symptomless autoimmune thyroiditis.^4–6^ Before circling back to these findings in relation to the Luo et al^1^ report in this journal, it is worth documenting other pertinent HPT axis findings in depression. These include: (a) blunted TSH response to intravenously administered TRH^7^ in approximately 25% of depressed patients, (b) elevated cerebrospinal fluid (CSF) concentrations of TRH^8^ and (c) reduced CSF transthyretin levels.^9^ Moreover, Cohen and colleagues^10^ reported that many depressed patients who are non-responsive to antidepressants with TSH levels above 3.5 mIU/mL respond when supplemented with T_4_. This finding is of interest for several reasons. First it suggests that the usual ‘normal range’ of TSH of 5–5.5 mIU/ml is not ‘normal’ for patients with depression. This fits with the observations reported many years ago that depressed patients with primary hypothyroidism do not respond to antidepressants, and that the classical physiological effects of antidepressants are not observed in hypothyroid rats. Moreover, Prange et al^11^ reported that T_3_ treatment accelerated the antidepressant effects of tricyclic antidepressants, and others reported T_3_ to be effective in converting antidepressant non-responders to responders.^12^ Finally, thyroid dysfunction has been reported to be a risk factor for the development of treatment-refractory depression.^13^

This commentary was prompted by the report by Luo et al.^1^ Their landmark study comprised 1718 first-episode drug-free patients who fulfilled DSM-IV criteria for major depression with Hamilton Rating Scale for Depression 17 scores greater than 23. A total of 438 patients (25.8%) had detectable TPOAb, and 303 patients (17.6%) had detectable TgAb. Not surprisingly, these patients had higher TSH concentrations compared with the major depressive disorder patients without positive anti-thyroid antibodies. A remarkable finding of this study concerns the history of suicide attempts in this population. In the overall major depression population, the lifetime suicide attempt rate was 20%. However, when the data were analysed with respect to the presence or absence of anti-thyroid antibodies, the difference between groups was robust – the rate of lifetime suicide attempts was 14% in those without the presence of thyroid antibodies and 34% in those with detectable thyroid antibodies. Those with high antibody titres exhibited suicide attempt rates of 44% (TgAb) and 54% (TPOAb), respectively.

Limited data are available on drug-free first-episode depressed patients, and the studies that have been reported include our own PReDICT study,^14^ which did not measure HPT axis function. There have been recent reports documenting the relatively high comorbidity of autoimmune disorders and depression,^15^ and the current findings confirm and extend these results with clear treatment implications. Normalisation of HPT function is clearly a prerequisite for optimal treatment of depression. Based on the present results, one can make the argument that in addition to measurement of TSH, anti-thyroid antibodies should also be measured in the evaluation of depressed patients. One also may wonder whether this finding is transdiagnostic and is not specific to depression but perhaps an integral contributor to suicide risk in other DSM-5 diagnoses.^16^ Surely, additional studies are warranted.
